# Exploring brushing and questionnaire data from a feasibility randomised control trial of a school-based smart, connected toothbrushing program

**DOI:** 10.1186/s12903-025-06581-3

**Published:** 2025-08-15

**Authors:** Michaela Goodwin, George Kitsaras, Momina Muzammil, Nicola Boothman, Juliana Gomez, Beulah Amulyavathi Gangaraju, Tanya Walsh

**Affiliations:** 1 Division of Dentistry, University of Manchester, Manchester, UK; 2 Colgate-Palmolive Company, Dental Health Unit, Williams House, Manchester Science Park, Manchester, M15 6SE UK

**Keywords:** Toothbrushing, Oral health, Technology, Connected devices, Text messages, School

## Abstract

**Background:**

Children’s oral health continues to be a key issue, with dental caries remaining one of the most prevalent non-communicable diseases. One of the main ways to prevent dental caries is to carry out adequate toothbrushing with fluoridated toothpaste. Connected or ‘smart’ toothbrushes can provide real-time feedback on brushing behaviour to individuals or be used to provide feedback to groups for example within a school setting. This study aimed to identify the engagement level of school children with a smart, connected toothbrush, explore brushing frequency, timing, coverage, and duration during the research period, and compare data to observed changes over time.

**Methods:**

Data on brushing behaviour were obtained from a feasibility cluster randomised controlled trial exploring smart, connected toothbrush use in a school-based brushing programme. Participants, aged 8–11 with access to a smart device, were recruited from six primary schools located in deprived areas of Manchester, England. Randomisation at the school level allocated participants to receive either a smart, connected toothbrush and text message intervention (intervention group) or the smart, connected toothbrush only (control group).

**Results:**

Of the 409 participants provided with a smart, connected toothbrush, 289 (71%) connected and used the device during the study. Of those who engaged with the connected devices, participants brushed at least twice a day on 48.2% of the days they brushed. On completed questionnaires, 78.8% of parents indicated their children brushed their teeth twice a day. Brushing frequency appeared to decrease over the weekends, particularly on weekend mornings.

**Conclusions:**

The results from this feasibility trial suggest that toothbrushing habits remain a fundamental area of importance, with a substantial proportion of children not brushing according to recommendations. Not all children connected or consistently used their devices during the study. Twice-daily brushing was infrequently recorded. Consideration must be given to the potential omission of brushing sessions, as the data may miss out sessions until the user syncs the brush with the app. Further research is needed to identify how frequency of brushing, particularly at the weekends, could be improved.

**Registration:**

The study was retrospectively registered at ISRCTN, registration number ISRCTN77803149 on 28/12/2023.

**Supplementary Information:**

The online version contains supplementary material available at 10.1186/s12903-025-06581-3.

## Background

Dental caries in children remains a major health burden and is closely linked to inadequate oral hygiene habits and consumption of free sugars [World Health Organisation, 2022]. Years of research have demonstrated that regular toothbrushing with fluoridated toothpaste can reduce the risk of dental caries by disrupting dental plaque and creating a beneficial environment to keep teeth healthy [1]. While manual toothbrushes are the most common tools used to perform this type of key oral hygiene behaviour, there has been increased interest in powered toothbrushes and smart, connected toothbrush devices to support good oral health. Smart, connected toothbrush devices are part of ‘Mobile health’ or ‘mHealth’ which refers to ‘medical and public health practices based on mobile devices such as mobile phones, patient monitoring systems, personal digital assistants, and other wireless devices’ [2]. These ‘smart’ connected toothbrushes can provide real-time feedback to users on toothbrushing duration, frequency and coverage.

School brushing programmes supporting optimal oral hygiene practices have been shown to improve children’s oral health and are cost-effective [3, 4]. However, they face barriers to implementation such as cross-infection control, issues in acquiring funding, additional classroom time required, time out of education and logistical challenges [5, 6].

In addition to connected technologies, there are other mHealth options, including the use of text messages through mobile phones. Short message service (SMS) has been increasingly used as a relatively inexpensive method of communication, can be used for communication across different age groups, is customizable, provides rapid, automated delivery and it’s acceptable to the public [7–10].

In this study, it was hypothesised that smart, connected toothbrush devices could be utilised within a primary school brushing programme, with additional text message support aimed at improving brushing behaviour by enabling individual feedback and providing classroom-level reinforcement through teacher engagement. This study explored both the feasibility and acceptability of this type of intervention (reported in a companion paper [11]), and presents indicative results from the toothbrushing data and the potential impact of the text message component.

### Aim & objectives

The overall aim of the study (the LEAPFROG study) was to explore the feasibility of a school-based toothbrushing programme using a smart connected toothbrush with young children across six primary schools (presented in the companion paper [11]. The aims and objectives of this article are to explore the toothbrushing and questionnaire data collected as part of the feasibility study.

## Methods

This feasibility cluster randomised controlled trial took place between March and August 2023. The study was approved by The University of Manchester Ethics Committee (Leapfrog study; 2022-15413-25761) and registered in ISRCTN (28/12/2023) (https://www.isrctn.com/ISRCTN77803149).

### Participants & sample size

Children in six schools across Greater Manchester aged 8 to 11 were invited to participate in the study. Inclusion criteria included access to home Wi-Fi and a smart device with internet connection compatible with the toothbrush and app (Apple iPhone (newer than 8) or iPad (newer than 5th generation) or Android device (running Android 6.0 or higher ); and no prior use of the smart, connected toothbrush. All families of children who were 8 to 11 years old within the consenting schools were invited to take part.

### Sample size

Six schools with double-form entry were invited to participate in the research. As a feasibility trial, this was a pragmatically chosen sample that would be sufficient in size to identify challenges principally concerning recruitment and feasibility of delivery of the intervention, and data collection.

### Interventions & randomisation

All participating children received a ‘hum by Colgate’ smart, connected toothbrush to use at home twice daily (morning and bedtime). The toothbrush captures information on coverage, duration and frequency of toothbrushing and communicates these data to children in real time via an accompanying app.

In addition to the toothbrush, teachers had access to a class-level dashboard that provided insights into their class’s toothbrushing performance (compared to other classes). They could also set challenges for students to help them improve toothbrushing behaviour or maintain strong practices if they were already performing well.

Three schools were allocated to additionally receive text message support (delivered to parents’ mobile phones). Text messages were directed at parents/caregivers and children and emphasized key oral health messages to motivate continued use of the toothbrush.

All intervention elements were delivered over a three-month follow-up period. Brushes were used at home directly by children with parental/caregiver supervision. Teachers received access to the weekly dashboard every Monday. In the text message trial arm, parents/caregivers received messages in a tapered approach, with more text messages sent at the start of the study and fewer towards the end. In total, each parent/caregiver received nine text messages over the course of the study.

### Randomisation

Allocation to trial arms was at the school level (simple randomisation [flipping a coin]), with participating schools receiving either the smart, connected toothbrush with text messages or the smart, connected toothbrush alone.

### Data collection

Separate questionnaires were administered to collect data on knowledge, attitude and practices (KAP) and on self-reported toothbrushing, oral hygiene practices, dietary practices and attitudes to oral health (see Appendix - Supplementary Materials 1). KAP questionnaires are frequently used in studies [12]. The version of KAP used in this questionnaire was adapted from a previous KAP used in previous studies by the authors [13]. The questionnaire on self-reported toothbrushing and oral hygiene practices included questions on whether they had brushed their teeth the day before, how many times, when they brushed, etc. See appendix 2). Questionnaire (Q) data were collected over five time points at baseline (Q1), one week after baseline (Q2), one month after baseline (Q3), two months after baseline (Q4) and 3 months after baseline (Q5). Data from the questionnaires were explored, concentrating on any changes from baseline to the final survey at 3 months. Toothbrushing data was collected from the hum by Colgate smart, connected toothbrushes.

Data collected by the connected toothbrushing device on date, time, coverage, and duration of each brushing event were explored to understand toothbrushing behaviours and whether these differed across weekdays and weekends, morning or evening. Toothbrushing behaviour was explored between the intervention group (who also received a text message intervention) and control group (where no text messages were provided) to present indicative results on the impact of the text message component. Engagement with the device was explored to understand if there were differences between those participants who used and synced the device across the duration of the study to those who did not. Finally, toothbrushing behaviour from the connected device was compared with self-reported toothbrushing behaviour to provide further insight into the reliability of these methods.

The data followed a multilevel (hierarchical) structure, with observations nested within a child, nested within a school. Analyses accounted for the multilevel structure and the lack of independence of observations. Participants were analysed according to the trial arm to which their school was randomised. As this was a feasibility trial, no formal hypothesis testing for effectiveness of the intervention was undertaken. The aim of the trial was to address existing uncertainties, specifically in terms of recruitment, retention, and engagement with the intervention rather than determining effectiveness.

## Results

**Fig. 1:**
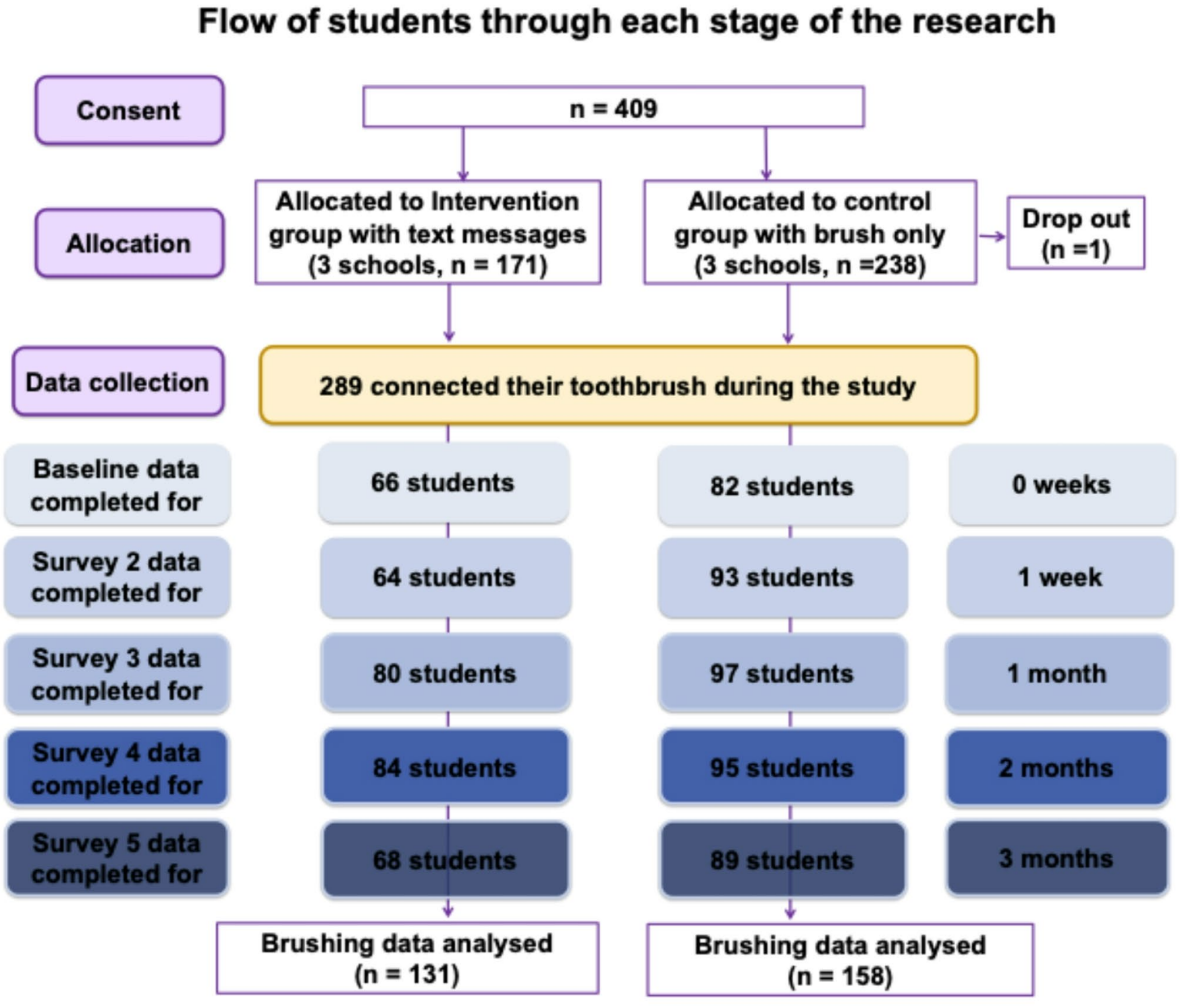
Participants’ flow diagram

Six schools were approached, with all six agreeing to participate. Seven hundred and fifty-three children were eligible to participate, of whom 409 (54%) consented and were provided with smart, connected toothbrush. Of those provided with a connected toothbrush, 289 connected and synced their toothbrush during the study (See Fig. 1).

### Sect. 1: questionnaire data

The data presented in this section provides an overall picture of the responses from this sample. The average age of participants was 9.7 years (SD 1.6) with 27 fathers and 121 mothers completing the baseline questionnaire (further analysis provided in Appendix Table 1, split by control and intervention). Table 1 details the descriptive statistics from the Brief Oral Health Survey. During the baseline period, 142 out of 145 respondents (97.9%) reported their child brushed their teeth the previous day, with similar numbers at follow-up. Out of this, 77.5% brushed both in the morning and at bedtime at baseline and 80.3% at the final follow-up. Among those who brushed their teeth at bedtime, 77.24% did not consume drinks or snacks afterwards at baseline, while this was 79.75% at the final follow-up. On a normal day, 62.76% ‘sometimes’ had sugary drinks/ snacks at baseline, whereas this was 74.05% at the final follow-up. When asked how important oral health was to their families at the time, 46.90% chose ‘most important’ at baseline and 67.09% chose the same at the final follow-up.

**Table 1:** Summary of the findings from the brief oral health survey

Variable	Q1	Q2	Q3	Q4	Q5
	Frequency (*N* = 409) (Percentage)	Frequency (*N* = 409) (Percentage)	Frequency (*N* = 409) (Percentage)	Frequency (*N* = 409) (Percentage)	Frequency (*N* = 409) (Percentage)
**Did your child brush his/her teeth yesterday?**					
No	1 (0.2)	-	1 (0.2)	2 (0.5)	1 (0.2)
Can’t remember/ Not sure	2 (0.5)	-	5 (1.2)	6 (1.5)	5 (1.2)
Yes	142 (34.7)	158 (38.6)	172 (42.1)	172 (42.1)	152 (37.2)
Missing	264 (64.6 )	251 (61.4 )	231 (56.5 )	229 (55.99)	251 (61.4 )
**(Conditional**,** if Yes in Q1) Was it in the morning**,** bedtime or both?**					
Morning	25 (17.6)	15 (9.5)	35 (20.4)	39 (22.7)	22 (14.5)
Bedtime	7 (4.9)	5 (3.2)	16 (9.3)	7 (4.1)	8 (5.3)
Both	110 (77.5)	138 (87.3)	121 (70.5)	126 (73.3)	122 (80.3)
**(Conditional if No or Can’t remember/Not sure in Q1) When was the last time your child brushed his/her teeth?**					
2 days ago	2 (0.5)	-	2 (0.5)	4 (1.0)	1 (0.2)
More than 2 days ago	-	-	-	-	1 (0.2)
More than a week ago	-	-	1 (0.2)	-	-
Can’t remember/Not sure	1 (0.2)	-	3 (0.7)	4 (1.0)	4 (1)
**If your child brushes their teeth at bedtime**,** does he/she have any drinks/snacks afterwards?**					
Yes, always	2 (0.5)	1 (0.2)	3 (0.7)	4 (1)	2 (0.5)
Yes, sometimes	27 (6.6)	30 (7.3)	29 (7.1)	43 (10.5)	28(6.9)
No, never	112 (27.4)	123(30.1)	140 (34.2)	131 (32.0)	126 (30.8)
Can’t remember / Not sure	4 (1.0)	4(1.0)	6 (1.5)	2 (0.5)	2 (0.5)
Missing	264 (64.6)	251 (61.4)	231 (56.5)	229 (56.0)	251 (61.4)
**On a normal day**,** does your child have any sugary drinks/snacks?**					
Yes, most days	48 (11.7)	43 (10.5)	52 (12.7)	47 (11.5)	36 (8.8)
Yes, sometimes	91 (22.3)	107 (26.2)	118 (28.9)	129 (31.5)	117 (28.6)
No, never	6 (1.5)	8 (2.0)	6 (1.5)	4 (1.0)	5 (1.2)
Can’t remember/ Not sure		-	2 (0.5)	-	-
Missing	264 (64.6)	251 (61.4)	231 (56.5)	229 (56.0	251 (61.4)
**On a scale of 1–10 (1- not important at all**,** 10- most important issue)**,** how important is oral health to your family right now?**					
1	1 (0.2)	1 (0.2)	-	-	-
2	-	2 (0.5)	-	1 (0.2)	-
3	-	-	-	-	1 (0.2)
4	-	1 (0.2)	-	-	-
5	2 (0.5)	4 (1.0)	1 (0.2)	1 (0.2)	2 (0.5)
6	6 (1.47)	-	1 (0.2)	4 (1.0)	-
7	16 (3.9)	9 (2.2)	4 (1.0)	3 (0.7)	6 (1.5)
8	29 (7.1)	27 (6.6)	37 (9.1)	28 (6.9)	21 (5.1)
9	23 (5.6)	25 (6.1)	24 (5.9)	36 (8.8)	22 (5.4)
10	68 (16.6)	89 (21.8)	111 (27.1)	107 (26.2)	106 (25.9)
Missing	264 (64.6)	251 (61.4)	231 (56.5)	229 (56.0)	251 (61.4)

Table 2 summarises responses from participants related to oral hygiene behaviour, including those who responded at both baseline and final follow-up. There was a 6.7% increase in participants who brushed their teeth at least twice a day from the baseline to the final follow-up. An increase (10.4%) was observed in self-reported fluoride toothpaste use within the household. The use of fluoride mouthwash twice a day remained relatively stable with 7.56–8.49% at baseline and final follow up respectively. Data was also collected on patients’ knowledge and attitudes related to dental health, summaries of these are presented in the Appendix (Appendix Tables 1 and 2).

**Table 2:** Summary of the results for questions related to practice

How often does your child brush his/her teeth?	Q1		Q5	
	Frequency (*N* = 106)	Percentage (100%)	Frequency (*N* = 106)	Percentage (100%)
Once a day	20	18.9	13	12.3
Twice a day	86	81.1	91	85.9
More than twice a day	-	-	2	1.9
**How often do you visit the dentist with your child?**				
Never	2	1.9	-	-
If there’s a problem	15	14.2	13	12.3
Once a year	21	19.8	17	16.0
Once every 6 months	56	52.8	66	62.3
Once every 3 months	12	11.3	10	9.4
**Do you use a fluoride toothpaste in your household?**				
No	8	7.6	3	2.8
Not sure / Don’t know	16	151	10	9.4
Yes	82	77.4	93	87.7
**Has your child ever had fluoride varnish at the dentist?**				
Yes	44	41.5	54	50.9
No	36	33.9	35	33.0
Not sure / Don’t know	26	24.5	17	16.0
**How often does your child use fluoride mouthwash?**				
Not at all	79	74.5	69	65.1
Once a day	18	16.9	25	23.6
Twice a day	8	7.6	9	8.5
More than twice a day	1	0.9	3	2.8
**Every time your child finishes brushing his/her teeth**,** what do you do immediately after?**				
Spit excessive toothpaste	58	54.7	67	63.2
Rinse with water	48	45.3	39	36.8
**On a normal night**,** after your child finishes brushing his/her teeth**,** do they have any food or drinks?**				
Yes, water	30	28.3	33	31.1
Yes, milk	3	2.8	4	3.8
Yes, food/drink other than water/milk	8	7.6	5	4.7
No	65	61.3	64	60.4

### Sect. 2: brushing data

#### Synced data from smart, connected devices 

Data in Table 3 shows how long participants used the smart, connected toothbrush device. It should be noted this does not necessarily mean daily use occurred but indicates the maximum recorded duration of use over this period of time. The data indicates whether the device was used for a single day, over a week, for at least one week but less than a month, for at least one month but less than two months, or for more than two months. Table 3 also provides the frequency of use by intervention/control groups. Both groups show a similar trend in participant engagement with the smart, connected toothbrush (used and synced), with 56% of participants in the control group and 52% of participants in the intervention group using and syncing the device for over two months. The data is also presented in a histogram showing the number of days brushed (Appendix Fig. 1) and the average brushing occurrence by days brushed (average brushes per day in Appendix Fig. 2). The majority of participants averaged between one or two brushing sessions per day.

**Table 3:** Duration of use during the study, by trial arm

Frequency of use	Control Number of users (%)	Intervention Number of users (%)	Total Number of users (%)
Over two months	89 (56%)	69 (52%)	158 (55%)
One to two months	24 (15%)	24 (18%)	48 (17%)
One week to one month	29 (18%)	18 (14%)	47 (16%)
2 to 7 days	13 (8%)	16 (12%)	29 (10%)
One day	3 (2%)	4 (3%)	7 (2%)
Total (engaged)	158 (100%)	131 (100%)	289 (100%)
Total engaged	158 (66%)	131(77%)	289 (71%)
Never engaged	80 (34%)	40 (23%)	120 (29%)
Total consented	238 (100%)	171 (100%)	409 (100%)

### Participant characteristics and engagement with the brush>

120 participants never connected, used or synced their toothbrush during the study. Short term engagers (*n* = 131) used the toothbrush but stopped using/syncing it within 2 months. Long term engagers (*n* = 158) used/synced the toothbrush over 2 months or for the duration of the study (Table 3). These three groups were compared against questionnaire responses with data on toothbrushing habits presented in Table 4. The majority of children in each of the subgroups reported brushing their teeth twice per day. Self-reported brushing habits were similar across engagement groups (80% non-engaged, 82% short-term, 76% long-term reported brushing twice daily). Appendix Tables 3, 4, and 5 include additional demographic and dental attendance data.

**Table 4:** Self-reported toothbrushing habits by engagement with a smart, connected toothbrush

How often does your child brush his/her teeth?	Non engaged 120 (29.3%)	Short term engager 131 (32.0%)	Long term engager 158 (38.6%)
Once a day	5 (20%)	8 (18%)	18 (24%)
Twice a day	20 (80%)	38 (82%)	58 (76%)
Total	25	46	76

Further analysis was conducted on a specific week during the study for those using the smart, connected toothbrush (the week was chosen as it did not fall on a school holiday and all participants had time to start using the toothbrush). For this week the toothbrushing data for weekdays and weekends were combined and compared.

Table 5 describes the mean count of toothbrushing occurrences over a single week, by time of day (morning or evening) and by trial arm (intervention and control). If all children brushed their teeth twice a day, we would expect to see weekday averages of 10 (5 mornings and 5 evenings), and weekend averages would be 4 (2 mornings and 2 evenings). Brushing was lowest on weekend mornings, with a mean of 0.90 (s.d. 0.94) in the control group and 0.96 (s.d. 0.99) in the intervention group. This suggests that when brushing sessions are missed, they are more likely to occur in the morning on weekends. Brushing occurrence was marginally higher for the intervention group on both the weekend and weekday.

**Table 5:** Weekday vs. Weekend brushing occurrence– AM and PM, intervention vs Control- for a week in May 2023

	Brushing occurrence	Mean (s.d.)	Min Max
Control	**Weekday total average (128)**	**7.05 (4.18)**	**0–35**
	Weekday am **average** (128)	3.82 (2.19)	0–16
	Weekday pm **average** (128)	3.15 (2.72)	0–18
	**Weekend average (128)**	**2.36 (1.72)**	**0–6**
	Weekend am **average** (128)	0.90 (0.94)	0–3
	Weekend pm **average** (128)	1.21 (1.08)	0–6
Intervention	**Weekday total average (100)**	**7.24(3.55)**	**0–19**
	Weekday am **average** (100)	3.79 (1.92)	0–9
	Weekday pm **average** (100)	3.39 (2.32)	0–10
	**Weekend total average (100)**	**2.58 (1.86)**	**0–10**
	Weekend am **average** (100)	0.96 (0.99)	0–4
	Weekend pm **average** (100)	1.47 (1.39)	0–7

In terms of duration of brushing, weekday brushing was longer in the control group (137.13 s) than in the intervention (126.64 s). However, brushing durations on weekends were similar across both groups (see Table 6).

**Table 6:** Weekday vs. Weekend toothbrushing duration weekday vs. Weekend - Intervention vs Control- for a week in May 2023

Duration of brushing (seconds)		Mean (s.d.)	Min Max
Control	Weekday **average** (127)	137.13 (31.57)	53–239
	Weekend **average** (101)	128.96 (41.35)	10–239
Intervention	Weekday **average** (99)	126.64 (37.60)	25–217.5
	Weekend **average** (82)	127.85 (35.91)	32–202.5

The coverage data was also explored by group. While only descriptive data is presented, it appears that coverage was similar between the control and intervention group, ranging between 80.27 and 84.49 (see Table 7).

**Table 7:** Weekday vs. Weekend brushing coverage - Intervention vs Control- for a week in May 2023

Brushing coverage*		Mean	Min Max
Control	Weekday **average** (127)	84.49 (15.75)	24–100
	Weekend **average** (101)	80.27 (21.07)	10–100
intervention	Weekday **average** (99)	80.40 (19.50)	24–100
	Weekend **average** (82)	82.02 (19.05)	24–100

*(the percentage coverage of teeth cleaned calculated by the connected device)

Data were analysed to determine whether participants brushed their teeth once or twice per day on average using the smart, connected toothbrush. This was calculated by assessing the number of brushing sessions recorded for each participant but only on days with recorded brushing data. Therefore, average brushing frequency was calculated based on days when participants had brushed at least once. The mean average was 0.48 (s.d. 0.29), suggesting for the days when brushing data was recorded, participants brushed twice daily (with their toothbrush active) on slightly fewer than half of the recorded days. When splitting this data by intervention and control group, it was observed that the intervention group (which received text messages) brushed twice a day with the connected toothbrush more often than the control (54% vs. 44%).

Further analysis of toothbrushing data is presented in Appendix Tables 6, 7, 8, 9 and 10 exploring brushing throughout the study duration and for the week May 15th to May 21^st,^ 2023.

The final analysis compared the toothbrushing data from the smart, connected device to the responses given by parents as to whether their child brushed their teeth the day before and whether this was in the morning or evening (Table 8). As not everyone connected and synced their toothbrushes, data could not be compared for days without recorded brushing. The absence of brushing data could reflect either a missed brushing session or failure to sync the device. Therefore, comparisons were made for those where there was both questionnaire data and at least one toothbrushing occurrence for the same day. Out of the 95 participants with both brushing data and questionnaire data, 71 (75%) showed agreement between the self-reported toothbrushing and toothbrushing data collected by the smart, connected toothbrushing device, with most reporting brushing their teeth twice a day.

Twenty-three participants out of 95(24%) reported brushing twice daily, while the connected device only registered one brushing event, either morning or evening, on those days. This discrepancy may have resulted from participants brushing without turning on the connected device or choosing to use a different toothbrush. These findings highlight potential limitations in the self-reported toothbrushing data that may not be accurately reported.

**Table 8:** Brushing data compared to self-reported brushing

		Brushing data			
		Brushed morning and Evening	Brushed morning only	Brushed evening only	Total
Questionnaire data	Brushed morning and Evening	59	14	9	82
	Brushed morning only	0	10	0	10
	Brushed evening only	1	0	2	3
	Total	60	24	11	95

## Discussion

The LEAPFROG study explored the feasibility of a school-based toothbrushing programme using a smart connected toothbrush. This article provides information on the participants within the study, their self-reported oral health behaviours and toothbrushing data collected through the smart connected toothbrush.

One of the main findings from the feasibility study was that the majority of children used the toothbrushing device at least once throughout the course of the study (71%). When comparing short-term, long-term, and non-users based on questionnaire responses, participants appeared similar in their reported tooth brushing frequency, with around 80% stating their child brushed twice daily. This indicates those connecting and using the toothbrush are not necessarily different from those who did not connect or use their toothbrush (although the results need to be treated with caution given the low numbers responding to questionnaires). Participant engagement and data synchronisation are important factors to consider if connected devices are to be used to monitor patient/participant behaviour in future studies or healthcare applications. Several variables may influence an individual’s decision to engage with such devices or the ability to synchronise their data. These could include but are not limited to, the device’s battery life and the ease of recharging the toothbrush, the usability of the accompanying app and the simplicity of syncing the data. Additionally, issues with connectivity, including weak or intermittent internet connectivity could hinder synchronisation attempts or the need for connected devices to sync to one particular phone/device [14–16]. Feedback from participants using connected toothbrushes who took part in qualitative interviews have highlighted some of these issues. One parent highlighted that their child always wanted to use their phone while brushing their teeth which interfered with a busy morning routine, and therefore they deleted the app. Another discussed that children may take the opportunity to play games, which was not the intended use. Furthermore, one parent noted that others had concerns about being constantly monitored [11].

While smart, connected toothbrushing devices are still a relatively new addition within oral health, some studies have provided information on users of connected devices and their engagement or synchronisation of devices with an app. One article looking at data from 1926 patients who had used a smart, connected toothbrushing device showed 73% of the sample were men, and there was some improvement in duration of brushing up to 60 days within this study [17]. Another feasibility study with 32 participants also indicated that the smart, connected toothbrush device had a high criterion validity for measuring oral health behaviours. However, retrospective self- reports from participants showed they recorded higher levels of toothbrushing frequency than recorded on the toothbrushing device [18]. Our feasibility study showed a similar result with 24% of respondents indicating their child had brushed their teeth in the morning and evening but the connected device recorded only one brushing session for that day.

When exploring brushing data between the control and intervention group there appeared to be a similar frequency of use with 56% of participants connecting and using the toothbrush for over two months in the control group, compared to 52% of participants in the intervention group. The intervention showed promise in encouraging toothbrushing behaviour twice a day with 54% brushing twice a day in the intervention group and 44% in the control group. This indicates that reminders through text messages could form part of an intervention to encourage adequate toothbrushing behaviour, but this would need to be explored further in a full randomised controlled trial. Previous research has also indicated that text messages can have a positive effect in improving bedtime routines for young children, including encouraging better toothbrushing practices [19]. The data presented in this article should be treated with caution, consideration must be given to the potential omission of toothbrushing sessions, as the data may exclude sessions if participants failed to sync the toothbrush with the app.

Further exploration of the data, looking at weekend and weekday brushing, revealed that children were less likely to have recorded toothbrushing sessions at the weekend, and particularly in the morning at the weekend. Therefore, future interventions may wish to target this period to encourage and support toothbrushing at weekends, particularly in the morning for school-aged children.

Data obtained from those who brushed and connected the toothbrushing device indicated that toothbrushing did not consistently occur twice daily, as recommended. There is limited research on data obtained from smart brushes. Previous studies have indicated that individuals do not brush their teeth for the recommended amount of time and have inconsistent toothbrushing patterns (although some of these studies had small sample sizes and data should be interpreted with caution) [20]. The use of the smart, connected toothbrush was not compared against a manual toothbrush in this study; however, previous studies have indicated smart, connected toothbrushes provide good education on brushing habits and could produce a plaque reducing effect [21].

The use of digital technologies to support good oral health has shown promise across a number of studies, from the use of mobile apps to encourage and motivate toothbrushing behaviour [22], to text messages to support parents/caregivers in establishing good oral health routines at bedtime [19] and the use of smart, connected toothbrushing devices to provide immediate feedback, supporting recommended toothbrushing behaviour [17, 21].

### Strengths and limitations

The data presented in this paper has been taken from a feasibility trial, and therefore the analysis was restricted to being more exploratory in nature. Given this the results should be interpreted with caution, as the study was not powered to detect significant differences between groups. Nonetheless, it provides early evidence of potential for the intervention. A third of participants did not connect the toothbrush, limiting access to data for that group. However, these participants had similar characteristics to those who did engage with the devices.

It should be noted that questionnaire response rate ranged from 35 to 45% across the five questionnaire time points. Therefore, again data should be treated with caution, as it may not represent the entire sample. Low questionnaire response rates are a common issue in oral health studies [23] and substantial efforts were made to increase and maintain response rates in this feasibility trial.

Despite the limitations of a feasibility study, this work has allowed for potential issues to be identified and the refinement of the study design to ensure future large scale studies involving smart, connected toothbrushing devices are successful. Future research could include strategies to support engagement with these devices, such as using devices with automatic syncing, and utilising multiple data capture methods that can be used to further cross reference data. Building on from data collected in this feasibility study, future research would require a larger and diverse sample, stratified to ensure results are representative.

## Conclusion

While taking the limitations of the study into account, the data does indicate that toothbrushing habits remain a key area of importance, with the possibility of a substantial proportion of children not achieving adequate toothbrushing (particularly at weekends when routines change) according to data collected using smart, connected devices.

Future research should consider how to quantify changes in children’s oral health, address loss of questionnaire completion and harness the opportunities this study provided including working closely with teachers and parents/caregivers to co-develop interventions which have the potential to positively impact children’s oral health.

## Electronic supplementary material

Below is the link to the electronic supplementary material.

Supplementary Material 1

Supplementary Material 2

Supplementary Material 3

## Data Availability

The datasets used and/or analysed during the current study are available from the corresponding author on reasonable request.
