# Supplementary material for: Exploring brushing and questionnaire data from a feasibility randomised control trial of a school-based smart, connected toothbrushing program
Source: BMC Oral Health. 2025 Aug 15;25:1330. doi: 10.1186/s12903-025-06581-3 (PMC12357393; doi:10.1186/s12903-025-06581-3)
Supplement: Supplementary file 1 — Supplementary Material 1 [file 12903_2025_6581_MOESM1_ESM.docx]

| **Knowledge** | |
| --- | --- |
| What is the most common disease in the world? | Diabetes  Heart disease  Dental cavities / decay  Gum disease  Arthritis |
| Do you think oral health relates to general health? | Yes  No  Not sure/Don’t know |
| Do you think tooth decay/cavities is avoidable? | Yes  No  Not sure/Don’t know |
| What are some of the signs and symptoms of dental diseases? | Toothache  Swollen puffy gums  Blood when brushing  Ear pain  All of the above  None of the above |
| What is the recommended strength of fluoride toothpaste for families? | 1350ppm to 1500ppm fluoride  Less than 1000ppm fluoride  Not sure/Don’t know |
| What is the recommended amount of toothpaste to use for children aged 3 to 6? | Smear  Pea sized  Full brush |
| **Attitudes** | |
| Do you agree or disagree that frequent consumption of sugar (candy, sweets, sugary drinks etc.) causes tooth decay? | Strongly Agree  Agree  Neither agree nor disagree  Disagree  Strongly Disagree  Not sure/Don’t know |
| Do you agree or disagree that dental decay and gum disease can be caused by plaque? | Strongly Agree  Agree  Neither agree nor disagree  Disagree  Strongly Disagree  Not sure/Don’t know |
| Do you agree or disagree that fluoride toothpaste strengthens teeth? | Strongly Agree  Agree  Neither agree nor disagree  Disagree  Strongly Disagree  Not sure/Don’t know |
| Do you agree or disagree that it is necessary to brush teeth frequently? | Strongly Agree  Agree  Neither agree nor disagree  Disagree  Strongly Disagree  Not sure/Don’t know |
| Do you agree or disagree that frequent visits to dental professionals are necessary? | Strongly Agree  Agree  Neither agree nor disagree  Disagree  Strongly Disagree  Not sure/Don’t know |
| **Practises** | |
| How often does your child brush his/her teeth? | Not at all  Once a day  Twice a day  More than twice a day |
| How often do you visit the dentist with your child? | Never  If there’s a problem  Once a year  Once every 6 months  Once every 3 months |
| Do you use a fluoride toothpaste in your household? | Yes  No  Not Sure/Don’t know |
| Has your child ever had fluoride varnish at the dentist? | Yes  No  Not Sure/Don’t know |
| How often does your child use fluoride mouthwash? | Not at all  Once a day  Twice a day  More than twice a day |
| Every time your child finishes brushing his/her teeth, what do you do immediately after? | Spit excess toothpaste  Rinse with water  Something else, please specify |
| On a normal night, after your child finishes brushing his/her teeth, do they have any food or drinks? | Yes, water  Yes, milk  Yes, food/drink other than water/milk  No |
| Brief Oral Health Survey | |
| Did your child brush his/her teeth yesterday? | Yes  No  Can’t remember/Not sure |
| (Conditional, if Yes in Q1)  Was it in the morning, bedtime or both? | Morning  Bedtime  Both |
| (Conditional if No or Can’t remember/Not sure in Q1)  When was the last time your child brushed his/her teeth? | 2 days ago  More than 2 days ago  More than a week ago  Can’t remember/Not sure |
| If your child brushes her teeth at bedtime, does he/she have any drinks/snacks afterwards? | Yes, always  Yes, sometimes  No, never  Can’t remember/Not sure |
| On a normal day, does your child have any sugary drinks/snacks? | Yes, most days  Yes, sometimes  No, never  Can’t remember/Not sure |
| On a scale of 1-10 (1-not important at all, 10-most important issue), how important is oral health to your family right now? | 1-10 scale |
