# Supplementary material for: Exploring brushing and questionnaire data from a feasibility randomised control trial of a school-based smart, connected toothbrushing program
Source: BMC Oral Health. 2025 Aug 15;25:1330. doi: 10.1186/s12903-025-06581-3 (PMC12357393; doi:10.1186/s12903-025-06581-3)
Supplement: Supplementary file 2 — Supplementary Material 2 [file 12903_2025_6581_MOESM2_ESM.docx]

**Appendix 1**

**Table 1:** Participant characteristics

| Child characteristics | Control | Intervention |
| --- | --- | --- |
| **Age** | 9.58 (1.39) | 9.81 (1.68) |
| **Ethnicity** |  |  |
| White | 56% (46) | 37% (24) |
| Asian British | 14% (12) | 41% (27) |
| Black British | 10% (8) | 8% (5) |
| Multiple | 11% (9) | 5% (3) |
| Other | 7% (6) | 8% (%) |
| Prefer not to say | 1% (1) | 1% (1) |
| **Child sex** |  |  |
| Male | 57% (47) | 31% (21) |
| Female | 43% (35) | 69% (36) |

Table 2 provides a summary of results for questions related to knowledge (see Appendix 2 for all of the questions asked during the study) ). The number of correctly answered questions was totalled for baseline and the 3-month questionnaire, with results provided in Table 6. The number of participants who answered all six questions correctly increased from 6.60% during the baseline to 12.26% during the final follow-up, while those who answered only 1 question correctly decreased from 4.72% to 0.94% suggesting an improvement in knowledge.

**Table 2: Summary of the results for questions related to knowledge**

| No. of questions (Knowledge) correctly answered | Q1 | | Q5 | |
| --- | --- | --- | --- | --- |
|  | Frequency (N=106) | Percentage | Frequency (N=106) | Percentage |
| All 6 | 7 | 6.60 | 13 | 12.26 |
| 5 | 28 | 26.42 | 35 | 33.02 |
| 4 | 41 | 38.68 | 33 | 31.13 |
| 3 | 21 | 19.81 | 16 | 15.09 |
| 2 | 4 | 3.77 | 8 | 7.55 |
| 1 | 5 | 4.72 | 1 | 0.94 |

Attitudes were examined using the following questions: Do you agree or disagree that frequent consumption of sugar (candy, sweets, sugary drinks, etc.) causes tooth decay?, Do you agree or disagree that dental decay and gum disease can be caused by plaque?, Do you agree or disagree that fluoride toothpaste strengthens teeth?, Do you agree or disagree that it is necessary to brush teeth frequently? And Do you agree or disagree that frequent visits to dental professionals are necessary? With individuals responding on a 5-point Likert scale from strongly agree to strongly disagree. When summarising the data ‘Strongly agree’ and ‘agree’ were grouped as ‘agree’, while strongly ‘disagree’ and ‘disagree’ were grouped as ‘disagree’. There was an increase in the number of participants who agreed across all five questions from 73.58% at baseline to 80.19% at the final follow-up (see Table 3).

**Table 3: Summary of the results for questions related to attitude**

| **Agreement/ Disagreement** | Q1 | | Q5 | |
| --- | --- | --- | --- | --- |
|  | Frequency | Percentage | Frequency | Percentage |
| Agreed across all 5 questions | 78 | 73.58% | 85 | 80.19% |
| Disagreed across all 5 questions | 0 | 0% | 0 | 0% |
| Neither agreed nor disagreed across all 5 questions | 0 | 0% | 0 | 0% |
| Not sure/Don’t know across all 5 questions | 0 | 0% | 0 | 0% |

Table 4, 5 and 6 show the self-reported responses to the questionnaire against the 3 groups according to engagement with the connected brush. No apparent difference in brushing behaviour, education or dental attendance were noted between the three groups.

**Table 4: Self-reported importance of brushing by engagement with Smart brush**

| Do you agree or disagree that it is necessary to brush teeth frequently | **Non engaged 120** (29.3%) | **Short term engager** 131 (32.0%) | **Long term engager 158** (38.6%) |
| --- | --- | --- | --- |
| Strongly agree/ agree | 24 (96%) | 44 (96%) | 75 (97%) |
| Neither agree nor dis | 0 | 1 (2%) | 0 |
| Disagree | 1 (4%) | 0 | 1 (1%) |
| Not sure / Don't know | 0 | 1 (2%) | 0 |
| Total | 25 | 46 | 76 |

**Table 5: Parent education by engagement with Smart brush**

|  | **Non engaged 120** (29.3%) | **Short term engager** 131 (32.0%) | **Long term engager 158** (38.6%) |
| --- | --- | --- | --- |
| GCSEs | 3 (12%) | 11 (24%) | 14 (18%) |
| A-levels | 4 (16%) | 2 (4%) | 9 (10%) |
| Vocational training g | 2 (8%) | 6 (13%) | 4 (8%) |
| University graduates | 6 (24%) | 14 (30%) | 26 (34%) |
| Postgraduate degree | 6 (24%) | 2 (4%) | 12 (16%) |
| Prefer not to say | 4 (16%) | 11 (24%) | 11 (14%) |
| Total | 25 | 46 | 76 |

**Table 6: Self-reported dental attendance by engagement with Smart brush**

|  | **Non engaged 120** (29.3%) | **Short term engager** 131 (32.0%) | **Long term engager 158** (38.6%) |
| --- | --- | --- | --- |
| Never | 1(4%) | 0 (0%) | 2 (3%) |
| If there's a problem | 4 (16%) | 7 (15%) | 7 (9%) |
| **Once a year** | 6 (24%) | 10 (22%) | 18 (24%) |
| Once every 6 months | 14 (56%) | 25 (54%) | 38 (50%) |
| Once every 3 months | 0 (0%) | 4 (9%) | 11 (14%) |
| Total | 25 | 46 | 76 |

**Figure 1: Number of days brushed**

**
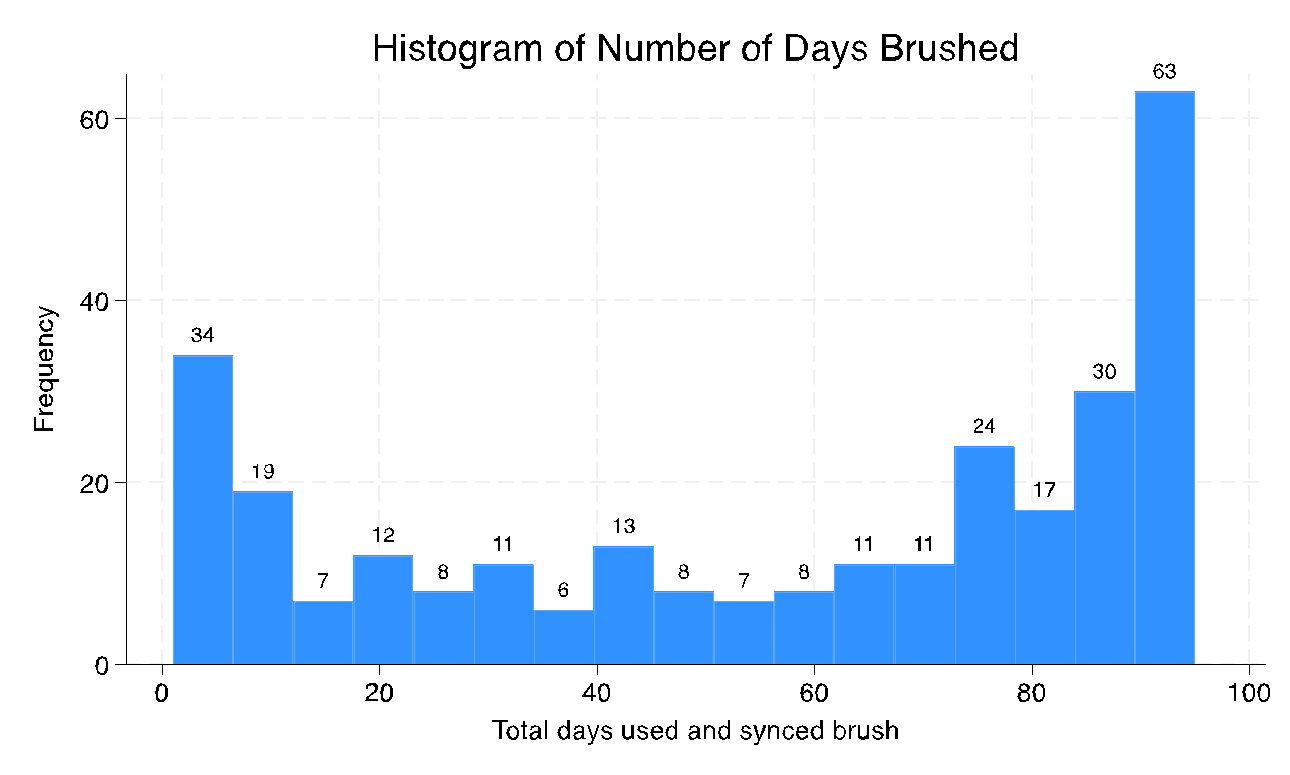
**

**Figure 2: Average brushes during the study by the total days brushed**

**
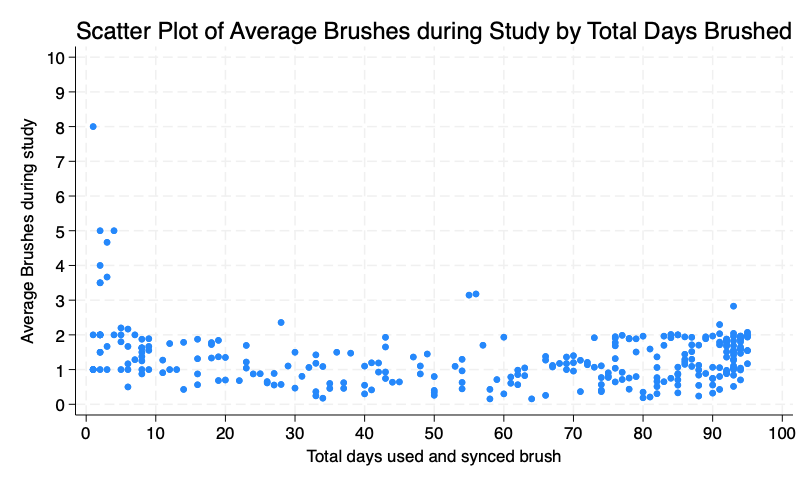
**

Table 7 shows the number of times on average a participant brushed during the week commencing 15th of May 2023. If participants brushed twice on each weekday there would be an average of 10 brushes, if they brushed twice each day on the weekend there would be an average of 4 brushes. It can be observed that on average a participant brushed 7.1 times on the weekdays and 2.5 times at the weekend, therefore carrying out 71% of the expected brushing occurrences during the weekdays and 63% of expected brushing occurrences at the weekend. This mirrors the overall data for brushing occurrences which demonstrates that brushing occurrences at the weekend may drop for some individuals.

**Table 7: Weekday vs Weekend brushing occurrence- May 2023**

| Brushing occurrence | Mean | s.d. | Min Max |
| --- | --- | --- | --- |
| Weekday ave (228) | 7.13 | 3.91 | 0 to 35 |
| Weekend ave (228) | 2.46 | 1.79 | 0 to 10 |

Table 8 describes the average brushing duration for the week of the 15th of May 2023 split between weekdays and weekend. As with brushing frequency, brushing duration decreases at the weekend compared to weekdays indicating responders on average spend 4 seconds less brushing their teeth when brushing on a weekend.

**Table 8: Weekday vs Weekend brushing duration**

|  | Mean | s.d. | Min Max |
| --- | --- | --- | --- |
| Weekday ave (226) | 132.54 | 38.91 | 10-239 |
| Weekend ave (183) | 128.46 | 38.91 | 10-239 |

Table 9 describes the average brushing coverage for the week of the 15th of May 2023 split between weekdays and weekend. As with brushing frequency and duration, the average coverage decreases at the weekend compared to weekdays indicating responders on average cover just 65% of the areas during the weekend compared to 81% during weekdays.

**Table 9: Weekday vs Weekend brushing coverage**

|  | Mean | s.d. | Min Max |
| --- | --- | --- | --- |
| Brushing coverage | |  |  |
| Weekday ave (226) | 82.66 | 17.57 | 24-100 |
| Weekend ave (183) | 81.07 | 20.15 | 10-100 |

The average duration of brushing observations across the study (taking into account the panel data is by child) is summarised in table 10. The average brushing duration is 128.4 seconds.

**Table 10**: **Average duration of brushing observations across the study**

|  | Mean | s.d. | Min - Max |
| --- | --- | --- | --- |
| Overall | 128.43 | 52.24 | 10 to 1261 |
| Between |  | 34.36 | 13 to 315.17 |
| Within |  | 44.16 | -88051 to 1283.34 |
| By Intervention | | | |
| Control (158) | 130.04 | 51.08 | 10 to 1261 |
| Intervention (131) | 126.65 | 53.43 | 10 to 956 |

The average coverage during brushing across the study (taking into account the panel data is by child) is summarised in table 11. The average coverage is 81.03% coverage

**Table 11**: **Average coverage of brushing observations across the study**

|  | Mean | s.d. | Min - Max |
| --- | --- | --- | --- |
| Overall | 81.03 | 25.34 | 0 to 100 |
| Between |  | 17.58 | 2 to 100 |
| Within |  | 20.44 | -16.64 to 143.34 |
| By Intervention | | | |
| Control (158) | 82.17 | 24.53 | 0 to 100 |
| Intervention (131) | 79.77 | 26.15 | 0 to 100 |
